# Task-Data Taxonomy for Health Data Visualizations: Web-Based Survey With Experts and Older Adults

**DOI:** 10.2196/medinform.9394

**Published:** 2018-07-09

**Authors:** Sabine Theis, Peter Wilhelm Victor Rasche, Christina Bröhl, Matthias Wille, Alexander Mertens

**Affiliations:** ^1^ Human Factors Engineering and Ergonomics in Healthcare Chair and Institute of Industrial Engineering and Ergonomics RWTH Aachen University Aachen Germany

**Keywords:** classification, data display, computer graphics, task performance and analysis, medicine, telemedicine, user/machine systems, human factors

## Abstract

**Background:**

Increasingly, eHealth involves health data visualizations to enable users to better understand their health situation. Selecting efficient and ergonomic visualizations requires knowledge about the task that the user wants to carry out and the type of data to be displayed. Taxonomies of abstract tasks and data types bundle this knowledge in a general manner. Task-data taxonomies exist for visualization tasks and data. They also exist for eHealth tasks. However, there is currently no joint task taxonomy available for health data visualizations incorporating the perspective of the prospective users. One of the most prominent prospective user groups of eHealth are older adults, but their perspective is rarely considered when constructing tasks lists.

**Objective:**

The aim of this study was to construct a task-data taxonomy for health data visualizations based on the opinion of older adults as prospective users of eHealth systems. eHealth experts served as a control group against the bias of lacking background knowledge. The resulting taxonomy would then be used as an orientation in system requirement analysis and empirical evaluation and to facilitate a common understanding and language in eHealth data visualization.

**Methods:**

Answers from 98 participants (51 older adults and 47 eHealth experts) given in an online survey were quantitatively analyzed, compared between groups, and synthesized into a task-data taxonomy for health data visualizations.

**Results:**

Consultation, diagnosis, mentoring, and monitoring were confirmed as relevant abstract tasks in eHealth. Experts and older adults disagreed on the importance of mentoring (χ^2^_4_=14.1, *P*=.002) and monitoring (χ^2^_4_=22.1, *P*<.001). The answers to the open questions validated the findings from the closed questions and added therapy, communication, cooperation, and quality management to the aforementioned tasks. Here, group differences in normalized code counts were identified for “monitoring” between the expert group (mean 0.18, SD 0.23) and the group of older adults (mean 0.08, SD 0.15; t_96_=2431, *P*=.02). Time-dependent data was most relevant across all eHealth tasks. Finally, visualization tasks and data types were assigned to eHealth tasks by both experimental groups.

**Conclusions:**

We empirically developed a task-data taxonomy for health data visualizations with prospective users. This provides a general framework for theoretical concession and for the prioritization of user-centered system design and evaluation. At the same time, the functionality dimension of the taxonomy for telemedicine—chosen as the basis for the construction of present taxonomy—was confirmed.

## Introduction

### Overview

Health care services are currently undergoing a digital transformation that is increasing the amount of clinical and personal health data. Data visualizations enable people to analyze and understand these data to make more informed decisions and to promote health-improving behavior [1-3]. Information and communication technology (ICT) development is the driving force behind the digitization of health services. In the 1990s, digital tools were differentiated from their analog counterparts with the prefix “e-.” Mail became email and commerce became e-commerce. Likewise, health became eHealth. The term describes all health services supported by ICT [4]. A definition covering all aspects of the term has not been achieved to date because it depends on ongoing technological development and diversity [5]. The major part of eHealth systems processes data to make it accessible to the user.

### Data Visualization

But what does the term data actually mean? Data—as the plural of the Latium *datum*—labels “factual information such as measurements or statistics used as a basis for reasoning, discussion, or calculation” [6]. Data results from a measurement [7]. In computer science, “data” is understood as machine-readable, digital representation of information encoded into character(s) (strings) following a syntax [8]. In order to abstract the information from data, it must be interpreted in a context of meaning; therefore, the user must be able to perceive and understand it [9]. Data visualizations are a way to make use of the effective visual perception channel to exchange information inherited in data [7]. By assigning graphical attributes to data, users can grasp data characteristics or identify new patterns [10-12]. As a graphical representation of data and statistical concepts, data visualizations particularly support decision making [13]. Data analysts, scientists, and statistical experts have been among the primary users of data visualization to date [14], but digitization of health services together with demographic change [15] and the recently observable shift toward patient empowerment are leading to an increase in the number of older adults without special background knowledge using data visualizations [16-21]. Accordingly, research on the visualization of health data is increasingly taking into account the perspective of older adults for design and evaluation [22-24].

### Task Models and Taxonomies

Before developers visualize data, they identify tasks relevant to users and data relevant to these tasks [25]. This ensures that visualization dashboards optimally support users in reaching their goals. In user-centered development, this is called task analysis as one method of the requirement analysis [26-28]. Thus, knowledge of visualization tasks is important for the selection or construction of suitable visual representations, at the same time it supports the empirical visualization evaluation during the selection of experimental tasks.

Tasks differ in their granularity and degree of abstraction [29,30]. For example, “curing a disease” is a domain task with low granularity (high-level task), whereas “compare a patient’s heart rate variability data to detect anomalies” describes a granular domain task (low-level task). Visualization tasks are determined by the user perspective [31] and numerous models exist to capture those inferring layers of data visualization tasks or processes [32-35]. Our work refers to Munzner’s model of nested layers [36]. Munzner’s nested model describes the procedure of data visualization design, starting with the investigation of domain tasks and data, because users have their own vocabulary to describe it. Subsequently, the domain problems have to be translated into abstract visualization tasks and data types as a vocabulary for data visualization. Data types in this context are defined by the kind of data to be visualized. In the third layer of Munzner’s nested model, visual encodings and interaction methods for data and task abstractions are developed so that corresponding algorithms can be developed at the innermost level. In this model, the output of one layer is the input for the subsequent one.

Abstract visualization tasks have often been listed alone or together with data types in the form of taxonomies [37]. Taxonomies are hierarchical structures originally used to classify organisms. Later, computer science used them to structure knowledge within knowledge-based systems or for software-testing research [38]. They provide conceptual clarity of a domain and categorize information for increased theoretical understanding. Another advantage is that taxonomies foster generalizability in empirical research if evaluation considers its tasks and data types [27,37,39-43]. Taxonomies also allow precise comparisons across different visualization tools and application domains. Work procedures can be analyzed using a domain-independent language, so that comparative analyses of tasks involving different visualization tools in different disciplines can be carried out [38,39]. A taxonomy is empirically built as the hierarchy of the concepts are classified by reason or measured similarity found in observed variables. A typology, in contrast, classifies various types that have equal characteristics and splits concepts into different types along at least two dimensions. It does not necessarily rely on empirical methods, and elements are less strictly reliant on the hierarchy as with a taxonomy.

An abstract task typology emerged from Munzner’s [36] nested model and was developed by Brehmer and Munzner [44]. Their typology includes a set of visualization tasks and data types with different levels of granularity (high level to low level), covering objectives on the “why dimension,” actions on the “how dimension,” and data types on the “what dimension.” We adopt their definition of data types: kind of data that can be visualized. The authors state that their typology is relevant for nearly all application domains. Thus, it might be assumed that it is also relevant for the eHealth domain. Empirical evidence has yet to be provided and it is one of the objectives of the investigation presented in this paper. The typology by Brehmer and Munzner partly overlaps with the data types from Shneiderman’s task-by-data-type taxonomy [37]. In a subsequently published article, Brehmer et al [45] characterized task sequences related to the visualization of dimensionally reduced data. Brehmer et al [46] also encourage detailed investigations of domain problems and tasks before the actual design and evaluation.

In the health and eHealth domain, taxonomies of general tasks have so far been applied to make concepts and their relation understandable. Furthermore, they are applied to differentiate ambiguous medical vocabulary [47-51]. For example, Bashshur et al [47] focused on the differentiation of different terms describing ICT-mediated health. The authors constructed a taxonomy of telemedicine by differentiating the subdomains telemedicine, telehealth, eHealth, and mHealth. They differentiated, as a part of the functionality dimension, the abstract tasks consultation, diagnosis, mentoring, and monitoring. The described taxonomy was built based on the expertise of the authors. A user study or literature review was not undertaken.

### Problem Statement

Previous literature illustrated the importance of task analysis with users for the description, evaluation, and creation of data visualizations. The problem is that if someone wants to develop a data visualization system, he or she must first find out which tasks the users consider relevant by means of user studies. Abstract visualization tasks as well as data and application-specific tasks play a role here. However, if all users had already been asked for their opinion on relevant tasks and data, developers could spare this time-consuming step of task analysis or at least parts of it.

In addition, it is almost impossible for scientists to adhere to the tasks that are relevant for users during an empirical evaluation of health data visualizations because this would require a separate study as a preanalysis of relevant user tasks. We believe not only developers may profit from using general tasks relevant to users as input for a more specific requirement analysis, but also researchers may consider them to select experimental tasks so that results from their evaluation become comparable and more generalizable across applications [52].

Although an extensive list of task taxonomies for data visualization exists, they are not suitable to lead developers and scientists to select tasks relevant to users because they are based on authors’ experience or on literature studies. They lack users’ perspectives. Another problem is that existing health taxonomies do not consider visualization-specific tasks and data, and taxonomies or typologies of abstract visualization tasks and data lack a definition of the domain problem and corresponding user tasks. Additionally, it remains unclear to what extent existing visualization task and data type classifications [44,47] are relevant to prospective eHealth users, who we—given the context of demographic change—consider to be older adults. Older adults are the ones who will use the future systems that developers can build based on the output of current research efforts. Furthermore, incidence, prevalence, and mortality are strongly age dependent. For this reason, the risk of developing age-dependent chronic diseases or psychological decline is rising. Thus, older adults are more likely to use eHealth systems than younger people are.

### Purpose of the Study

With this study, we want to make a first step toward generalizable results of user-centered task analysis, so that results are valuable to as many developers and researchers in the domain of eHealth as possible. Therefore, the purpose of this study is to construct a taxonomy of abstract domain and abstract visualization tasks and data types. To the best of our knowledge, we are the first to investigate the relation between abstract visualization tasks and data types in the eHealth context and thus the first to create a taxonomy that has domain relevance but remains general across different eHealth applications. In contrast to existing work, we construct the task taxonomy with the help of prospective eHealth users (older adults), so that it can foster the understanding of the user, the users’ tasks, and the users’ domain understanding in order to become a language among researchers from different domains. In this regard, the study will answer the following questions:

1. Which abstract eHealth tasks do older adults consider relevant for eHealth systems?
2. Which abstract visualization tasks and data types do older adults consider relevant for medical consultation, diagnosis, mentoring, and monitoring?
3. Does the rating from older adults differ from that of eHealth experts?

## Methods

### Study Design

We devised a structured cross-sectional study with a nonrandom sample to collect data from prospective eHealth users (older adults) and eHealth experts.

### Participants

Prospective eHealth users were targeted by focusing on participants older than 50 years because they are the ones who will use the future systems that developers can build based on the output of current research efforts. Furthermore, incidence, prevalence, and mortality are strongly age dependent with risks rising, for example, for chronic diseases or cognitive and physical decline [53]. Finally, yet importantly, the handling and perception of technology or relevant tasks is strongly influenced by the experiences individuals have made with technological artifacts during their lives. The so-called technology generations represent a major influence here [54]. We wanted to focus on the third group, called the “generation of technology spread” aged between 53 and 67 years. Thus, a perspective uninfluenced from existing digital technology could be taken, so that developers and researchers are able to orient toward the users’ native needs.

We additionally approached eHealth experts to provide evidence for the validity of the answers from the group of older adults. Basically, the expert’s answers served as baseline information to show if and where background knowledge has an impact or not.

### Recruitment

The sampling procedure was nonprobabilistic and purposive and respondents were selected based on their voluntary willingness to participate [55,56]. To approach described experimental groups with differing eHealth background knowledge, different recruitment channels were applied. For control purposes, the background knowledge was queried with only one question instead of with a battery of standard eHealth literacy questions. This way we could keep the questionnaire as short as possible.

We sent the link to an online survey to eHealth experts from our existing network in Germany. The survey was presented in the German language. Then we automatically extracted additional expert email addresses from the e-health-com webpage, where readers recommend experts. Editors of the website review the propositions and, if they consider a person an expert, the website lists them all alphabetically and provides one profile page per expert containing the name and position together with a short description, contact information, and affiliation description. We extracted all email addresses of experts automatically from the website by means of a Python script. We subsequently sent the link to the online questionnaire to 70 of these experts by email. Of these 70, 24 came from eHealth industry companies either as chief executive officer of a company selling eHealth products or as consultant active in the domain, and 40 came from research institutes working with information technology in the health sector. The remainder were medical experts from various domains or politics.

Older adults were selected by a clickworker platform [57] according to the demographic characteristic of being older than 50 years. Only participants who stated they were 50 years or older were able to access the survey. The link to the survey was displayed as a task on the website of the platform. At the end of the survey, participants were provided with an individually generated password. The participants had to provide the password to be credited with money to their accounts. We opted for a fee of €3 for completing the survey, which is relatively high because it was an abstract, and probably a more difficult subject, for participants not familiar with it.

### Survey Instrument

Data were collected via an online survey. The rationale for the use of an online questionnaire was that abstract tasks could be investigated by means of a sample larger than would have been possible with observations or qualitative in-depth interviews. The survey instrument was programmed and made available on a website using SurveyMonkey software [56].

The survey was introduced as a study “improving digital health care systems according to user needs” and consisted of five questions (for introduction text and survey questions see Multimedia Appendix 1). All participants were informed about the duration of the survey, data storage, and the leading investigator. After an introductory page, individual pages with one question per screen were displayed. The participant was able to skip to the next question, but was not able to return to the previous one. On all survey pages, it was ensured that the user could see all answer options without the need for scrolling. The answer options for all questions contained a checkbox with the label “no answer” (n/a) to keep track if the participant just forgot, or could not, or did not want to provide an answer. Therefore, answering a question was not mandatory in order to not frustrate participants and to collect as much information as the participants wanted to provide.

Subsequent to the introductory page, experts and older adults were asked to list medical tasks that they considered relevant for health systems (see question #1 in the questionnaire in Multimedia Appendix 1). This was presented as an open question to not restrict the participants’ views and to collect as much input as possible, while excluding priming effects that may occur if a list of possible answers was given. The second question was a closed question asking users to rate the relevance of consultation, diagnosis, mentoring, and monitoring for eHealth on a five-point Likert scale (question #2). Subsequently, participants had to rate the importance of abstract visualization tasks (“why” dimension) [44] for each task in Bashshur et al’s functionality dimension (consultation, diagnosis, mentoring, and monitoring; question #3). Finally, the relevance of data types [37,44] for consultation, diagnosis, mentoring, and monitoring [47] was assessed by means of a checkbox matrix (see question #4) and the background knowledge was assessed by a five-point Likert scale (see question #5). The survey was tested by two independent examiners with regard to wording and technical functionality.

### Data Collection

Data were collected between February 29 and March 14, 2016, from a sample of eHealth experts, and on November 16, 2016, from a sample of people older than 50 years without experience in eHealth. The time interval between the elicitation with experts and the one with older adults was because of prolonged approval for using the clickworker portal.

In total, 163 unique individuals visited the website of our Web-based survey. Identifying individuals was ensured by using the IP address and cookie function. Of these 163 visitors, 65 never started the survey. In total, 98 visitors participated in the survey; the participation rate was 74.4%. The average time spent completing the survey was 16 minutes 52.96 seconds.

### Analysis

The open-ended answer (see Multimedia Appendix 1, question #1) was first analyzed in terms of the overall word frequencies with the help of MaxQDA software [58]. Word frequencies were computed and all occurring words were listed. After the elimination of stop words (eg, in, on, where, why), the resulting word list was manually scanned for activities and tasks. The most frequent tasks became an item within a hierarchical dictionary. The dictionary items were named and structured referring to Bashshur et al’s [47] functionality dimensions. Each dimension (consultation, diagnosis, mentoring, or monitoring) became an item in the dictionary as a child of the root node eHealth tasks as soon as it occurred in the word list. Tasks from the word frequency list that did not have a “part of” relation with existing categories were considered the child of the root node eHealth tasks—and thus a sibling of consultation, diagnosis, mentoring, or monitoring. Two experienced qualitative analysts conducted the manual scanning of tasks and the structuring of the dictionary independently. The two analysts then discussed differing opinions when they assigned an item from the frequency list to the dictionary or when they sorted the dictionary and then implemented a common solution. Then, each item (task) in the dictionary contained a list of synonyms from the word frequency list. For example, the dictionary item “prevention” contained the words from the frequency list: prevention, explanatory work, hospital stay, tertiary prevention, avoidance, and care.

Subsequently, the MaxQDA software automatically coded all words in the answer texts with the item name from the dictionary they were assigned to. As a result, the dictionary contained code frequencies per dictionary item, which added up from lower to higher structural levels. Consequently, lower levels meant lower code frequencies. Code frequencies of items on higher levels were a sum of the item’s own code frequency together with the code frequencies of all subordinate levels (child items).

For the statistical computation of code count differences among the two experimental groups, the root level was included up to a maximum of the third level down the hierarchical structure. For statistical computation, the code frequencies were normalized with the total number of words the participants gave in their answer. Therefore, for the analysis of the answers on the closed questions, we used SPSS software, version 22 (IBM Corp, Armonk, NY, USA). To compare answers of eHealth experts and older adults, *t* tests for independent samples and chi-square tests were calculated, both at a significance level of .05.

### Taxonomy Construction

Our taxonomy for eHealth visualization tasks and data included the perspective of both experimental groups: the tasks and data types that they agreed on and group differences. Individual items have been ranked from top to bottom, according to task relevance. The more important an element was, the higher it was positioned.

Taxonomy construction started with abstract eHealth tasks resulting from closed question # 2 (see Multimedia Appendix 1) that participants rated as relevant. Tasks resulting from the open question #1 that were not already referred to by results from question #2 were then added as siblings. Subsequently, we added data types from question #3 and the top-ranked abstract visualization tasks resulting from question #4 to each of the four abstract eHealth tasks from question #2 (consultation, diagnosis, mentoring, and monitoring).

Group differences were reflected by the outline of a taxonomy item. Thick outlines of items illustrated that there were no significant differences between older adults and eHealth experts, whereas dotted outlines were significantly more important for experts and thin-outlined items were significantly more important for older adults.

Abstract visualization tasks that users most frequently considered relevant were included in the taxonomy. To determine the most relevant, we initially ranked all visualization tasks based on the amount they were considered relevant (“relevance count”). Then we computed the difference between the relevance counts of consecutive tasks (“relevance count difference”). The relevance count difference measure served to intensify the differentiation between relevant and nonrelevant abstract visualization tasks. This reinforcement of the distance between abstract visualization tasks became necessary in order to not include too many of them.

All abstract visualization tasks mentioned more frequently than the one with the second-biggest relevance count difference to its successor were included in the taxonomy. For example, the relevance of visualization tasks for consultation exhibited the two biggest differences between perceive information and search information (relevance count difference=8) and query information and lookup information (relevance count difference=6). In this case, query information and all tasks with higher total frequency exhibiting no group differences became part of the taxonomy.

### Approval and Informed Consent

The Ethics Committee at RWTH (Rheinisch-Westfälische Technische Hochschule) Aachen Faculty of Medicine, Germany, authorized this study and its ethical and legal implications in its statement EK236/16.

## Results

### Participants

A total of 98 people participated: 47 eHealth experts and 51 older (≥50 years) adults. The mean age of the eHealth experts was 42.3 (SD 7.3) years, and the mean age for the older adults was 55.8 (SD 5.9) years. The eHealth knowledge of the eHealth experts was comprehensive (8/47, 17%) or very good (39/47, 83%), whereas for the older adults it was neutral (15/51, 29%), low (27/51, 53%), or very low (9/51, 18%).

### Relevance of Medical Tasks

The most frequently mentioned eHealth tasks in open-answer texts were cooperation, consultation, mentoring, monitoring, documentation, communication, therapy, and quality management (see Table 1). In contrast to Bashshur et al [47], diagnosis constituted a subtask of therapy. Of all therapy subtasks, it had the highest frequency, followed by treatment. Extensions of the original taxonomy could be made concerning the scope of eHealth tasks, their structure, their validity, and their user relevance.

Group differences in the code count were computed on the first and second level except for the functionality dimension subconcept therapy, which together with all its child nodes reached a triple-digit code count. All normalized frequencies showed a normal distribution. An independent sample *t* test was conducted—as the normalized code frequencies were continuous variables not originating from predefined categories—to compare the code count of tasks and all child nodes of “therapy” between older adults and the eHealth experts. There was a significant difference in the scores for the code frequency of monitoring for eHealth experts (mean 0.18, SD 0.23) and older adults (mean 0.08, SD 0.15; *t*_96_=2.43, *P*=.02). Monitoring was more important for experts than for older adults.

**Figure 1:**
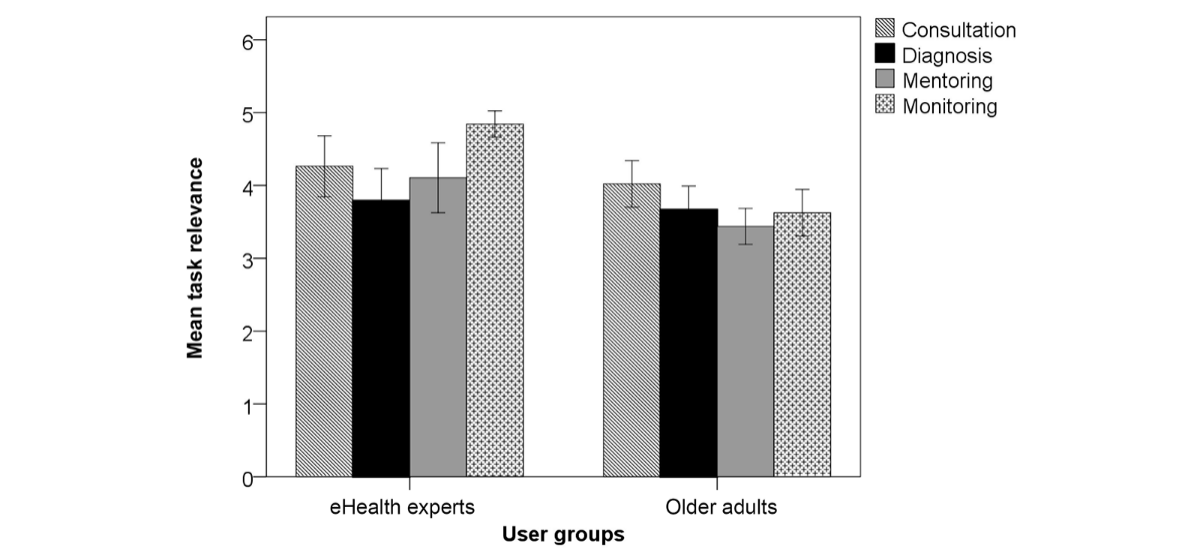
Mean relevance of individual eHealth tasks according to older adults and eHealth experts. Task relevance rated from 0=very low to 5=very high. Error bars represent 95% CI.

The closed question on eHealth task relevance revealed that across groups the relevance of eHealth systems for consultation and monitoring was most frequently considered very high. We received 70 valid answers, of which 51 came from older adults and 19 from the eHealth expert group (Figure 1, Table 2).

**Table 1:** Task relevance based on code frequencies in open answers in older adults and eHealth experts.

eHealth tasks and subtasks		Word frequencies in older adults, n	Word frequencies in experts, n	Total, N
Cooperation		10	14	24
**Consultation (total)**		39	66	105
	Consultation	25	37	62
	Physician-physician	3	18	21
	Physician-patient	10	11	21
	Physician-pharmacist	1	0	1
**Monitoring (total)**		42	82	124
	Monitoring	23	48	71
	Patient condition	0	1	1
	Observation	3	0	3
	Interpreting data	2	1	3
	Data transmission	3	4	7
	Data collection	6	8	14
	Patient behavior	0	1	1
	Medication	0	1	1
	Therapy progression	0	4	4
	Vital signs	0	13	13
	Health condition	1	0	1
	Wound surveillance	1	0	1
	Identifying saliences	3	1	4
	Patient condition	0	1	1
**Mentoring (total)**		22	21	43
	Mentoring	11	11	22
	Assistance	5	2	7
	Health suggestions	0	2	2
	Instructions	6	2	8
	Education	0	4	4
**Documentation (total)**		12	11	23
	Documentation	6	7	13
	Symptoms	1	0	1
	Surgery	1	0	1
	Wound documentation	0	2	2
	Experience reports	2	0	2
	Patient information	2	2	4
**Communication (total)**		44	52	96
	Communication	25	29	54
	Data handling/review	7	16	23
	Information search	10	3	13
	Date arrangement	2	3	5
	Billing	0	1	1
**Therapy (total)**		98	165	263
	Therapy	54	95	149
	Home care	2	5	7
	Diagnosis	30	37	67
	After treatment	2	4	6
	Treatment	6	12	18
	Rehabilitation	2	3	5
	Prevention	2	9	11
Quality		1	3	4

**Table 2:** Relevance of eHealth tasks in older adults (older) and eHealth experts(expert).

eHealth task	Very low, n (%)			Low, n (%)			Neutral, n (%)			High, n (%)			Very high, n (%)			Total, N	
	Experts	Older	Experts		Older	Experts		Older	Experts		Older	Experts		Older	Experts		Older
Consultation	0 (0)	3 (6)	1 (5)		1 (5)	2 (11)		8 (16)	7 (37)		19 (37)	9 (47)		20 (38)	19		51
Diagnosis	0 (0)	2 (4)	2 (11)		7 (14)	4 (21)		7 (14)	9 (47)		21 (41)	4 (21)		14 (28)	19		51
Mentoring	0 (0)	2 (4)	2 (11)		2 (4)	2 (11)		20 (39)	7 (37)		21 (41)	8 (42)		3 (6)	19		48
Monitoring	0 (0)	3 (6)	0 (0)		5 (10)	0 (0)		11 (22)	3 (16)		20 (38)	16 (84)		12 (24)	19		51

**Figure 2:**
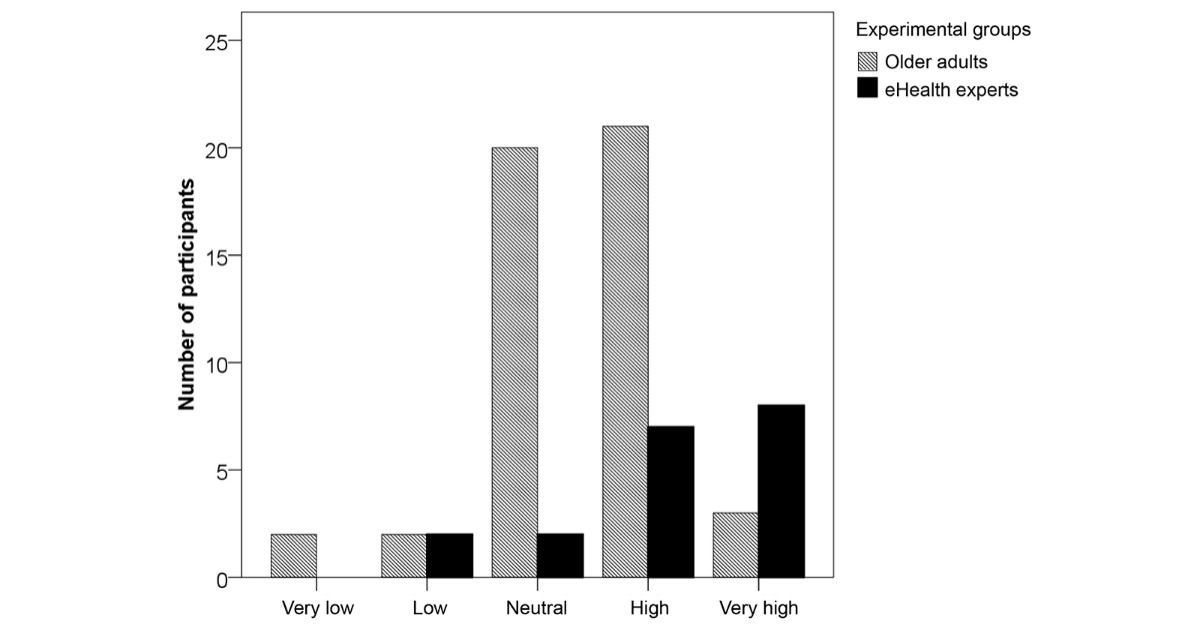
Relevance of eHealth for mentoring.

**Figure 3:**
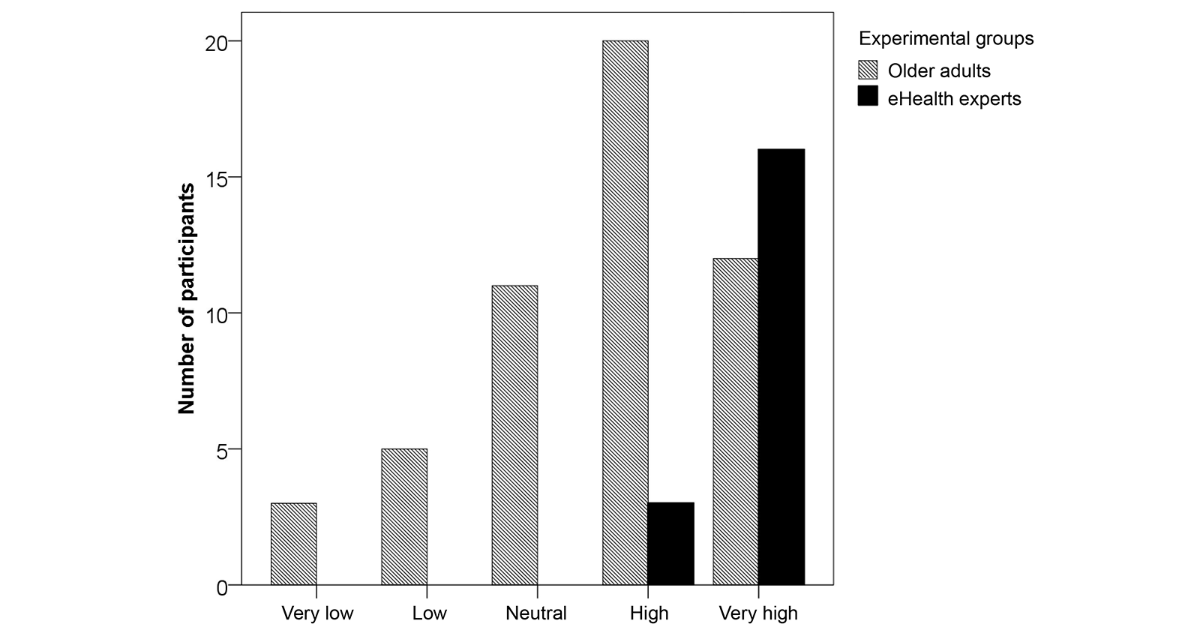
Relevance of eHealth for monitoring.

A chi-square test of independence was performed to examine the relation between relevance counts and user group (older adults, eHealth experts). The relation between these variables was highly significant for mentoring (χ^2^_4_=14.1, *P*=.002) and monitoring (χ^2^_4_=22.1, *P*<.001). Descriptive values of significant relevant differences are illustrated in Figures 2 and 3.

### Relevance of Abstract Visualization Tasks

The tasks perceive, search, record, present, annotate, and query information were most important for consultation across the whole sample. For diagnosis, the priorities were perceive, discover, search, locate, and identify information. For mentoring, the most relevant abstract visualization tasks were present, compare, generate, browse, and select information, whereas monitoring included generate, encode, consume, select, browse, and compare information (Table 3).

### Relevance of Data Types

A chi-square test of goodness-of-fit revealed that data types relevant to consultation, diagnosis, mentoring, and monitoring differed significantly between groups for most data types. The five most relevant data types were included into the taxonomy.

Additionally, the data type relevance for eHealth tasks (Tables 4-7) exhibited few cases in which the relevance frequency exceeded half the number of valid answers. The most relevant data types for consultation were quantitative data, nominal data, time-dependent data, points in time, and single values.

For diagnosis, time-dependent data, quantitative data, anomalies, single values, and points in time were most important across groups. Mentoring exhibited time-dependent data, rates of change, single values, quantitative data, and points in time as the most relevant data types.

According to the participants, monitoring required time-dependent data as the most important data type, followed by temporal patterns, rates of change, and quantitative data, and single values. In total, time-dependent and quantitative data could be numbered among the types with the highest frequencies.

**Table 3:** Abstract visualization tasks relevant for consultation, diagnosis, mentoring, and monitoring in older adults and eHealth experts (N=68).

Visualization task		N	Older adults, n (% from group)	eHealth experts, n (% from group)	χ^2^_1_	*P* value
**Consultation**						
	Perceive information	53	39 (74)	14 (26)	0.3	.75
	Search information	41	27 (66)	14 (34)	4.6	.05
	Record information	41	31 (76)	10 (24)	0.1	.88
	Present information	40	27 (68)	13 (33)	2.9	.15
	Annotate information	40	29 (73)	11 (28)	0.3	.78
	Query information	39	29 (74)	10 (26)	0.1	.88
**Diagnosis**						
	Perceive information	47	34 (72)	13 (28)	0.6	.55
	Discover information	47	34 (72)	13 (28)	0.6	.45
	Search information	46	33 (2)	13 (28)	0.8	.37
	Locate information	43	33 (77)	10 (23)	0.2	.66
	Identify information	42	34 (81)	8 (19)	2.0	.15
**Mentoring**						
	Present information	36	24 (67)	12 (33)	2.8	.09
	Compare information	36	27 (75)	9 (25)	0.0	.99
	Generate information	33	23 (70)	10 (30)	1.0	.33
	Browse information	33	22 (67)	11 (33)	2.4	.12
	Select information	33	22 (67)	11 (33)	2.4	.16
**Monitoring**						
	Generate information	38	25 (66)	13 (34)	3.9	.05
	Encode information	37	33 (89)	4 (10)	8.7	.01
	Consume information	35	21 (60)	14 (40)	8.7	.01
	Select information	35	26 (74)	9 (26)	0.2	.89
	Browse information	34	24 (71)	10 (29)	0.7	.40
	Compare information	34	24 (71)	10 (29)	0.7	.40

**Table 4:** Data types relevant for consultation.

Data types	N	Older adults, n (% from group)	eHealth experts, n (% from group)	Total relevant, n (% from N)	χ^2^_1_	*P* value
Quantitative data	92	32 (71)	15 (32)	47(43)	14.1	.001
Time dependent	95	27 (56)	13 (32)	40 (42)	8.0	.001
Single values	91	25 (57)	13 (28)	38 (42)	7.9	.01
Points in time	92	28 (62)	9 (18)	37 (40)	17.7	.001
Nominal data	79	19 (59)	13 (28)	32 (40)	7.9	.01
Ordinal data	77	16 (53)	13 (28)	29 (38)	5.1	.03
Time spans	92	22 (49)	7 (15)	29 (32)	12.3	.001
Temporal patterns	90	19 (43)	9 (17)	28 (31)	6.6	.01
Time intervals	91	20 (46)	7 (15)	27 (30)	10.2	.001
Anomalies	88	19 (46)	8 (17)	27 (31)	8.9	.01
Outlier	82	14 (30)	7 (39)	22 (27)	1.7	.21
1-D data	74	9 (33)	12 (26)	21 (28)	0.5	.59
Distributions	79	14 (44)	7 (15)	21 (27)	8.1	.01
Rates of change	91	21 (30)	10 (28)	31 (34)	7.1	.01
Groups	69	8 (15)	8 (17)	16 (23)	3.2	.12
Time sequences	88	14 (34)	5 (11)	15 (17)	7.2	.01
Synchronizations	82	9 (26)	6 (13)	15 (18)	2.3	.16
Multidimensional data	75	5 (18)	10 (21)	15 (20)	0.1	.78
Clusters	68	6 (29)	5 (11)	11 (16)	3.4	.08
2-D data	76	3 (10)	7 (15)	10 (21)	0.3	.73
3-D data	74	2 (7)	8 (17)	10 (21)	1.4	.31
Tree data	73	7 (27)	7 (15)	14 (19)	1.6	.23
Network data	70	3 (13)	7 (15)	10 (14)	0.1	>.99
Graphs	78	7 (14)	9 (19)	16 (21)	0.1	.78

**Table 5:** Data types relevant for diagnosis.

Data types	N	Older adults, n (% from group)	eHealth experts, n (% from group)	Total relevant, n (% from N)	χ^2^_1_	*P* value
Time dependent	95	37 (77)	16 (34)	53 (56)	17.8	.001
Quantitative data	92	32 (71)	16 (34)	48 (52)	12.7	.001
Anomalies	88	36 (88)	12 (25)	48 (55)	34.3	.001
Single values	91	32 (73)	13 (28)	45 (50)	18.5	.001
Points in time	92	32 (71)	11 (23)	43 (47)	21.0	.001
Outliers	82	24 (69)	14 (30)	38 (46)	12.1	.001
Time intervals	91	27 (61)	10 (21)	37 (41)	15.1	.001
Time spans	92	27 (60)	9 (19)	36 (39)	16.1	.001
Nominal data	79	24 (75)	11 (23)	35 (44)	20.5	.001
Rates of change	91	23 (52)	11 (23)	34 (37)	8.1	.01
Temporal patterns	90	26 (61)	7 (15)	33 (37)	20.1	.001
Time sequences	88	24 (59)	7 (15)	31 (37)	18.3	.001
Ordinal data	77	18 (60)	11 (23)	29 (38)	10.5	.01
2-D data	76	17 (59)	12 (26)	29 (38)	8.3	.01
1-D data	74	17 (63)	11 (23)	28 (38)	11.0	.001
Graphs	78	19 (61)	9 (19)	28 (36)	14.4	.001
3-D data	74	15 (56)	11 (23)	26 (35)	7.8	.01
Distributions	97	17 (53)	9 (19)	26 (33)	10.0	.01
Multidimensional data	75	12 (43)	12 (26)	24 (32)	2.4	.13
Groups	96	15 (68)	8 (17)	23 (33)	17.7	.001
Clusters	86	13 (62)	9 (19)	22 (32)	12.1	.001
Synchronizations	82	13 (37)	5 (11)	18 (22)	8.2	.01
Net data	70	8 (35)	9 (19)	17 (24)	3.0	.23
Tree data	73	10 (39)	6 (13)	16 (22)	6.5	.02

**Table 6:** Data types relevant for mentoring.

Data types	N	Older adults, n (% from group)	eHealth experts, n (% from group)	Total relevant, n (% from N)	χ^2^_1_	*P* value
Time dependent	95	18 (38)	13 (28)	31 (33)	1.1	.38
Rates of change	91	19 (43)	12 (26)	31 (34)	3.1	.08
Single values	91	23 (52)	8 (17)	31 (34)	12.6	.001
Quantitative data	92	17 (38)	12 (26)	29 (32)	1.6	.26
Points in time	92	18 (40)	11 (23)	29 (32)	2.9	.12
Time spans	92	19 (42)	9 (19)	28 (32)	5.8	.02
Temporal patterns	90	17 (40)	11 (23)	28 (31)	2.0	.12
Anomalies	88	18 (40)	9 (19)	27 (31)	6.3	.02
Nominal data	79	13 (41)	13 (28)	26 (33)	1.5	.33
Time intervals	91	14 (32)	10 (21)	24 (36)	1.3	.34
Time sequences	88	16 (39)	8 (17)	24 (27)	5.3	.03
Graphs	78	15 (48)	8 (17)	23 (30)	8.8	.01
Ordinal data	77	9 (30)	13 (28)	22 (29)	0.1	.99
1-D data	74	12 (44)	9 (19)	21 (29)	5.4	.03
Clusters	68	10 (48)	9 (19)	19 (28)	5.	.02
2-D data	76	10 (35)	8 (17)	18 (24)	3.0	.10
Distributions	79	10 (31)	8 (17)	18 (23)	2.2	.18
3-D data	74	8 (30)	9 (19)	17 (23)	1.1	.39
Synchronizations	82	12 (34)	5 (11)	17 (21)	6.8	.01
Multidimensional data	79	10 (36)	7 (15)	17 (21)	4.3	.05
Outlier	82	10 (29)	7 (15)	17 (21)	2.3	.02
Tree data	73	10 (39)	6 (13)	16 (22)	6.5	.02
Groups	69	8 (36)	8 (17)	16 (23)	3.2	.12
Net data	70	8 (35)	7 (15)	15 (21)	3.6	.07

**Table 7:** Data types relevant for monitoring.

Data types	N	Older adults, n (% from group)	eHealth experts, n (% from group)	Total relevant, n (% from N)	χ^2^_1_	*P* value
Time dependent	95	22 (46)	15 (32)	37 (40)	1.9	.21
Temporal patterns	90	22 (51)	13 (28)	35 (39)	5.2	.03
Rates of change	91	21 (48)	12 (26)	33 (36)	4.8	.03
Quantitative data	92	14 (31)	17 (26)	31 (34)	0.3	.66
Points in time	92	20 (44)	11 (23)	31 (34)	4.6	.05
Single values	91	18 (41)	13 (28)	31 (34)	1.8	.19
Time spans	92	19 (43)	11 (23)	30 (33)	3.7	.08
Graphs	78	20 (65)	10 (21)	30 (39)	14.6	.001
Synchronizations	82	21 (60)	8 (17)	29 (35)	16.2	.001
Multidimensional data	75	16 (57)	13 (28)	29 (39)	6.4	.02
Time intervals	91	16 (36)	12 (26)	28 (31)	1.3	.36
2-D data	76	18 (46)	9 (19)	27 (36)	14.4	<.001
Time sequences	88	17 (42)	10 (21)	27 (31)	4.5	.06
Outliers	82	14 (40)	12 (26)	26 (32)	1.9	.23
Anomalies	88	17 (42)	9 (19)	26 (30)	5.2	.03
3-D data	74	14 (53)	11 (23)	25 (34)	6.2	.02
Distributions	79	16 (50)	9 (19)	25 (32)	8.4	.01
Nominal data	79	15 (47)	9 (19)	24 (30)	6.9	.01
Ordinal data	77	9 (30)	12 (26)	21 (27)	0.2	.79
Groups	69	11 (50)	10 (21)	21 (30)	5.8	.02
1-D data	74	10 (37)	10 (21)	20 (27)	2.2	.18
Clusters	68	10 (48)	9 (19)	19 (28)	5.8	.02
Tree data	73	11 (42)	7 15	18 (25)	6.8	.01
Net data	70	9 (39)	8 (17)	17 (24)	4.1	.07

### Task-Data Taxonomy for eHealth Visualizations

**Figure 4:**
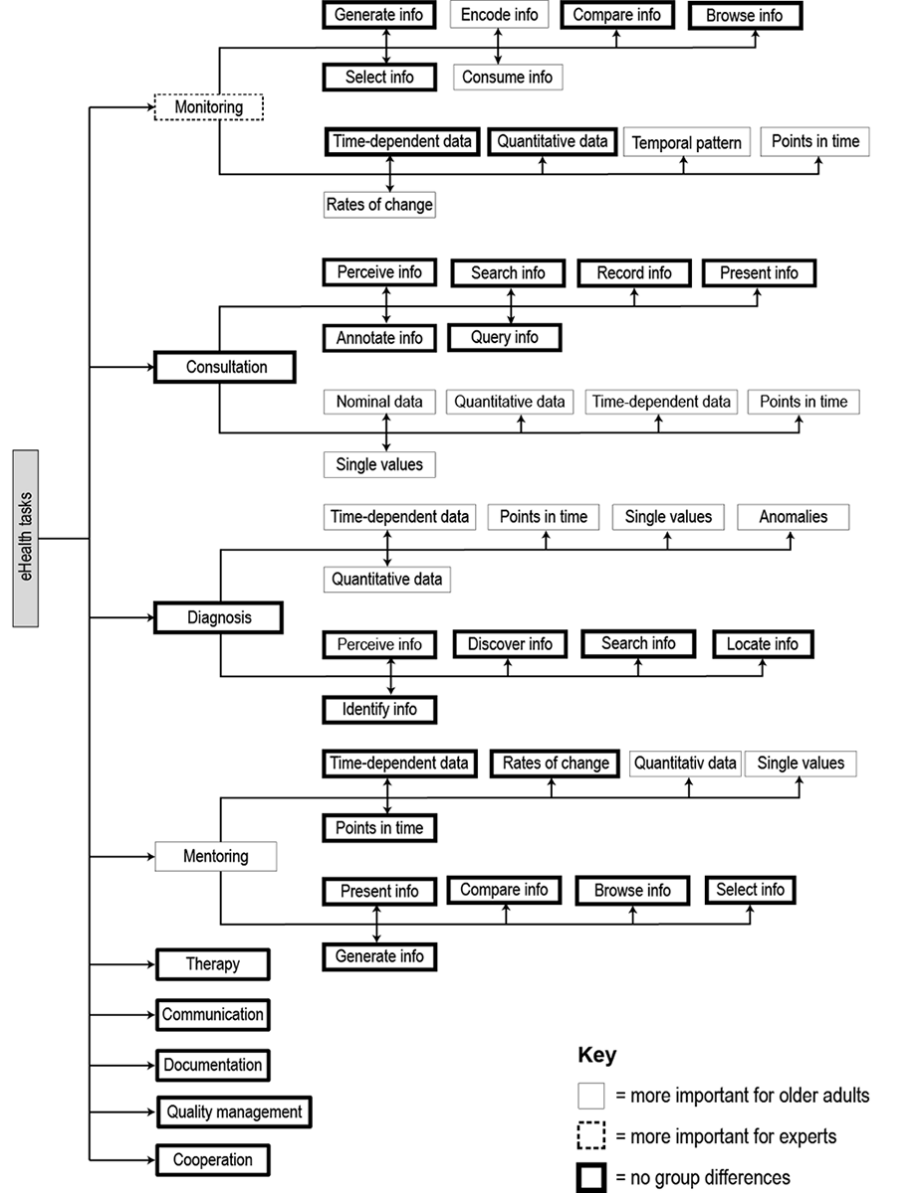
The eHealth visualization task-data taxonomy.

The task-data taxonomy for eHealth visualizations was constructed as described in the Methods section of our paper. It shows which health tasks are important for them and which abstract visualization tasks and data types are relevant for the abstract health tasks monitoring,” consultation, diagnosis, and mentoring. Group differences within the taxonomy are marked with different outline characteristics of the taxonomy item, which gives it higher meaning (dotted line=experts, thin line=older adults, thick line=no difference). It is striking that all relevant abstract visualization tasks were considered relevant by both groups, so there were no significant differences in relevance (see Figure 4).

## Discussion

### Principal Findings

This section offers a discussion and interpretation of the results regarding the task analysis of eHealth and visualization tasks and the corresponding data types across the two experimental groups: eHealth experts and older adults. We additionally elaborate on the limitations of our findings and describe future work.

The eHealth experts’ answer texts led to a total of 244 codes, whereas 155 codes could be derived from the older adults’ answer texts. Here, therapy was most frequently mentioned across the whole sample with a number of 263 counts including all subtasks (see Table 1), followed by monitoring (n=124), consultation (n=105), communication (n=96), mentoring (n=43), documentation (n=23), and quality management (n=4). Monitoring was seen differently across user groups: it was significantly more important to the experts than to the older adults. Diagnosis was found to be the most frequently mentioned subtask of therapy followed by treatment, prevention, home care, aftertreatment, and rehabilitation. The tasks at the second level were cited less frequently. The therapeutic tasks users considered most important were diagnosis and treatment. The former is important for both groups, whereas medical or eHealth experts cited treatment and prevention twice or more frequently. Collecting data as well as monitoring of vital data were the most commonly mentioned subtasks of monitoring in the participants’ opinions. Similar to the task at the first level (monitoring), there is a clear group difference with a focus on the maximum in the expert group.

It appears that code frequencies are relatively low compared to the whole sample size. This can be explained by the short, keyword-like answers most participants gave. For example, the sample group of 98 mentioned monitoring only 61 times. Considering that each code count cannot even be exclusively assigned to one person, results from a starting point for taxonomy construction requires future iterative improvement with a larger sample size as well as a validation of the hierarchical arrangement of individual elements [59,60].

Results of the open answers confirm the relevance of the functionality dimension within the taxonomy of telemedicine and that given task classification could be extended by the tasks therapy, cooperation, documentation, communication, and quality management. Results regarding confirmation of the functionality dimension of the taxonomy of telemedicine are in line with the results of our previous work [61].

The abstract eHealth tasks of the functional dimensions formed the root nodes of our taxonomy by their later assignment to abstract visualization tasks and data types; therefore, the validity of the analysis of the open and uninfluenced responses was validated by directly querying their importance with five-point Likert scales. The analysis of those closed questions on the relevance of the tasks consultation, diagnosis, mentoring, and monitoring supported results from the qualitative content analysis of open questions. Here, both user groups considered monitoring and diagnosis the most important eHealth tasks. The discrepancy between groups regarding the importance of the task monitoring was replicated as well.

Against the background of current work on the development of eHealth applications [9,62-66], we would have expected monitoring to be the most relevant eHealth task. The results of code count frequencies do not match this expectation. Because the results of previous studies on the investigation of health-related information need are consistent with the fact that, for adults older than 50 years, diagnosis is the most important information during the maintenance and administration of their personal health [67], it can be assumed that older adults regard the relevance of individual eHealth tasks less from a technology perspective. Tasks that are important for personal health have increased importance for older adults.

The background knowledge of older adults regarding the technical possibilities of eHealth systems differs from that of eHealth experts. Conventional constant monitoring or medical control has been less important to laypeople because it might be unclear to them that when it comes to continuous monitoring of sensor data, technical systems are often more accurate and stable at monitoring patients than medical personnel. The mental model that seems to influence the answer—even if the term was explained at the beginning of the survey—is more strongly characterized by health-relevant tasks users know from their everyday life, where the extensive introduction of digital monitoring systems is still pending in Germany.

At first sight, one might suspect this is a problem for the utility of the developed task-data taxonomy. However, this is only the case if one assumes that our taxonomy should precisely represent the tasks currently present in systems. However, the aim of the taxonomy is—as described at the outset—an increase in the user-centricity of future systems. For our taxonomy, it is not important which tasks and data actually exist, but which are relevant for prospective users, so that systems developed based on presented taxonomy have the greatest possible value. However, users’ perceptions of the relevance of individual tasks and data types are of great importance.

The question on the relevance of abstract visualization tasks was not answered by nearly a third of the participants (30/98). Whether a lack of knowledge or a lack of motivation is responsible cannot be determined on the basis of the data. Because 75% of older adults and only 25% of experts answered the question, despite experts having higher background knowledge, motivation seems to be more likely an influencing factor here.

We also assume that the eHealth systems including such visualizations are not available to some participants. Therefore—as in the case of the abstract eHealth tasks—the results identify potential areas where data visualizations could enable experts or patients to be supported in the corresponding medical task.

Our ranking of general eHealth tasks supports the general understanding of the application context of eHealth and eHealth visualizations from the perspective of prospective users (older adults). Visualizations that support those general domain tasks are expected to have a stronger impact. The intention here is not to invite visualization researchers to contribute designs to the eHealth domain, but to identify potential for the application of visualizations within eHealth systems, an aspect that has often been overlooked.

### Transfer of Knowledge

The presented results add to the increasing number of papers that target hierarchical task structures to establish a common vocabulary and understanding of visualization tasks and data [68,69]. This work goes beyond that by considering the context of eHealth including the perspective of the prospective user and synthesizing their input in the form of eHealth task-data taxonomy. In this way, eHealth system developers and researchers can use it as an orientation during requirement analysis or as a guideline for the definition of experimental tasks in visualization evaluation experiments.

### Limitations

We consider the described eHealth task-data taxonomy as provisional and subject to validation in the field. In addition, we only tracked the subjectively perceived knowledge about eHealth systems, so participants might lack familiarity with abstract data types or task-data taxonomies or they may not be familiar with online surveys and interactive Web tools such as those used for our Web survey. Thus, we are not able to quantify participants’ familiarity with concepts mentioned in this study and this may have influenced our findings. Familiarity with abstract data types and visualization tasks and styles common to the survey website would have likely reduced some of the barriers participants might have experienced.

Furthermore, as with subjective methods in general, results are limited in a way that they reflect the perspective and mental model of the participants together with their experiences. But observations will have the drawback that achievable sample sizes are much smaller, so that the results are hardly generalizable to the whole eHealth domain.

Additional limitations of our study lie in the selective sample caused by using an online questionnaire. People who are familiar with technology are more likely to answer the questionnaire than people who are not. Additionally, the older adults were paid, whereas the experts were not. This leads to different motivations between the two groups, which could be an influencing variable. This might have been the reason why the numbers of completed answers varied in the expert group over the length of the questionnaire (more were answered at the beginning than at the end).

### Conclusion

We successfully constructed a task-data taxonomy for eHealth data visualizations by providing a general description of tasks and data useful for health data visualizations. We have shown that semantic approaches [26] are feasible to generally perform task analysis. Furthermore, the results empirically validated and ranked Brehmer and Munzner’s [44] typology of abstract visualization tasks, as well as the functionality dimension of Bashshur et al’s [47] taxonomy of telemedicine. Time-dependent data and searching for information within visualizations of monitoring data had the highest relevance across user groups.

## Appendix

Multimedia Appendix 1 Text survey introduction and questionnaire.

## Abbreviations

ICT: information and communication technology
