# Supplementary material for: Task-Data Taxonomy for Health Data Visualizations: Web-Based Survey With Experts and Older Adults
Source: JMIR Med Inform. 2018 Jul 9;6(3):e39. doi: 10.2196/medinform.9394 (PMC6056737; doi:10.2196/medinform.9394)
Supplement: Multimedia Appendix 1 [file medinform_v6i3e39_app1.pdf]

Appendix

Text Survey Introduction for EHealth Experts and Older Adults (German)

Sehr geehrte/r Teilnehmer/in,
Wir führen eine Umfrage zu eHealth Systeme durch, um dadurch Forschungsfragen beantworten und Patientenversorgung verbessern zu können. Dabei würden wir gerne wissen, welche medizinischen Aufgaben durch digitale Gesundheitssysteme Ihrer Meinung nach verbessert werden können und welche Informationen und Daten für einzelne Aufgaben Ihrer Ansicht nach benötigt werden. EHealth bedeutet, dass unter Einsatz digitaler Informationstechnologie (Computer, Smartphone, Internet etc.) medizinische Dienste angeboten werden. Die Umfrage sollte nur fünfzehn Minuten in Anspruch nehmen, und Ihre Antworten sind gänzlich anonym. Sie können nur einmal an der Umfrage teilnehmen. Mit einem Sternchen (*) markierte Fragen sind obligatorisch.

Text Survey Introduction for EHealth Experts and Older Adults (English)

Dear participants,
We are conducting a survey on eHealth systems to answer research questions and improve patient care. We would like to know which medical tasks can be improved by eHealth systems and what information and data are required for individual tasks. EHealth means that medical services are offered using digital information technology. The survey should take only fifteen minutes, and your answers are completely anonymous. You can only participate in the survey once. Questions marked with an asterisk (*) are obligatory.

1. **What medical tasks and activities do you think profit the most from eHealth systems? (mention as many as possible)**

2. **Please assess the benefit of eHealth for the following tasks:**

	Very High	High	Neutral	Low	Very Low	n. A.
Consultation	☐	☐	☐	☐	☐	☐
Diagnose	☐	☐	☐	☐	☐	☐
Mentoring	☐	☐	☐	☐	☐	☐
Monitoring	☐	☐	☐	☐	☐	☐

3. **Indicate what data you think is necessary for the following tasks:**

	Consultation	Diagnosis	Mentoring	Monitoring	k. A.
Quantitative data	☐	☐	☐	☐	☐
Ordinal Data	☐	☐	☐	☐	☐
Nominal data	☐	☐	☐	☐	☐
1-dimensional data	☐	☐	☐	☐	☐
2-dimensional data	☐	☐	☐	☐	☐
3-dimensional data	☐	☐	☐	☐	☐
Time-dependent data	☐	☐	☐	☐	☐
Points in time	☐	☐	☐	☐	☐
Time intervals	☐	☐	☐	☐	☐
Time spans	☐	☐	☐	☐	☐
Temporal patterns	☐	☐	☐	☐	☐
Rates of change	☐	☐	☐	☐	☐
Time sequences	☐	☐	☐	☐	☐
Multidimensional data	☐	☐	☐	☐	☐
Tree data	☐	☐	☐	☐	☐
Net data	☐	☐	☐	☐	☐
Single values	☐	☐	☐	☐	☐
Outlier	☐	☐	☐	☐	☐
Distributions	☐	☐	☐	☐	☐
Anomalies	☐	☐	☐	☐	☐
Cluster	☐	☐	☐	☐	☐
Groups	☐	☐	☐	☐	☐
Graphs	☐	☐	☐	☐	☐

Questionnaire

Questionnaire

4. Which of the following informational tasks (vertical) are relevant for the given eHealth tasks (horizontal)?

	Consultation	Diagnosis	Mentoring	Monitoring	n. a.
Perceive information	☐	☐	☐	☐	☐
Present information	☐	☐	☐	☐	☐
Discover information	☐	☐	☐	☐	☐
Generate information	☐	☐	☐	☐	☐
Verify information	☐	☐	☐	☐	☐
Enjoy information	☐	☐	☐	☐	☐
Search information	☐	☐	☐	☐	☐
Lookup information	☐	☐	☐	☐	☐
Locate information	☐	☐	☐	☐	☐
Browse information	☐	☐	☐	☐	☐
Explore information	☐	☐	☐	☐	☐
Identify information	☐	☐	☐	☐	☐
Query information	☐	☐	☐	☐	☐
Compare information	☐	☐	☐	☐	☐
Summarize information	☐	☐	☐	☐	☐
Encode information	☐	☐	☐	☐	☐
Manipulate information	☐	☐	☐	☐	☐
Select information	☐	☐	☐	☐	☐
Navigate through information	☐	☐	☐	☐	☐
Arrange information	☐	☐	☐	☐	☐
Change information	☐	☐	☐	☐	☐
Filter information	☐	☐	☐	☐	☐
Aggregate information	☐	☐	☐	☐	☐
Introduce information information	☐	☐	☐	☐	☐
Annotate information	☐	☐	☐	☐	☐
Import information	☐	☐	☐	☐	☐
Derive information	☐	☐	☐	☐	☐
Record information	☐	☐	☐	☐	☐

5. My knowledge in digital health systems is....

- ☐ Very extensive
- ☐ Extensive
- ☐ Neutral
- ☐ Low that
- ☐ Very low
- ☐ n. a.
